# Correction: Prospective follow-up of New York City residents with e-cigarette, or vaping product use-associated lung injury—2020–2021

**DOI:** 10.1371/journal.pone.0341781

**Published:** 2026-01-26

**Authors:** Kathryn M. Tannert Niang, Aviva B. Grasso, Indira Debchoudhury, Dena Bushman, John P. Jasek, Monique A. Fairclough, Katherine R. Van Oss, Shadi Chamany, Kendall D. LaSane, Sharraine M. Franklin, Achala K. Talati

The second affiliation for the fourth author is incorrect. Dena Bushman is only affiliated with #2: Division of Scientific Education and Professional Development, CDC, Epidemic Intelligence Service, Atlanta, Georgia, United States of America.
