# Supplementary material for: Prospective follow-up of New York City residents with e-cigarette, or vaping product use-associated lung injury—2020–2021
Source: PLoS One. 2025 Apr 30;20(4):e0304918. doi: 10.1371/journal.pone.0304918 (PMC12043135; doi:10.1371/journal.pone.0304918)
Supplement: S2 File — (PDF) [file pone.0304918.s002.pdf]

U.S. Department of Health and Human Services
Centers for Disease Control and Prevention

Print Date: 6/11/24

**Title:**	Follow-up surveillance on New York City residents who have been discharged from the hospital for e-cigarette, or vaping, product use associated lung injury (EVALI).
**Project Id:**	0900f3eb81addd30
**Accession #:**	CSELS-EWBG9-2/20/20-ddd30
**Project Contact:**	Dena Bushman
**Organization:**	CSELS/DSEPD/EWBG9
**Status:**	**Project In Progress**
**Intended Use:**	**Project Determination**
**Estimated Start Date:**	02/20/2020
**Estimated Completion Date:**	02/20/2021
**CDC/ATSDR HRPO/IRB Protocol #:**	
**OMB Control #:**	

Determinations

Determination	Justification	Completed	Entered By & Role
HSC:<br>**Does NOT Require HRPO Review**	Not Research - Public Health Surveillance<br><br>*45 CFR 46.102(l)(2)*	3/10/20	Sinha_Saswati (sds2) CIO HSC
	**Exclusion:** Information collection not conducted or sponsored by Federal government		

PRA:<br>**PRA does not apply**	*Justification:* NYC DOHMH, and in collaboration with NYS DOH will lead the information collection to conduct surveillance of NYC residents.	3/10/20	Sinha_Saswati (sds2) OMB/PRA

Description & Funding

Description

**Priority:**	Standard
**Date Needed:**	02/28/2020
**Priority Justification:**	
**CDC Priority Area for this Project:**	Not selected
**Determination Start Date:**	02/20/20
**Description:**	Under the direction of NYC DOHMH, and in collaboration with NYS DOH, we will collect follow-up data on NYC residents who have been discharged from the hospital for EVALI. We will use telephone surveys conducted every 3 months for a year, and if needed, following appropriate consent, will obtain additional medical record information related to follow-up care. The following health and social information since hospital discharge will be collected: 1. Respiratory symptoms 2. Non-respiratory symptoms, including GI symptoms and behavioral health symptoms 3. Functional status, pre- and post-acute injury, including impact on school or work 4. Health behaviors 5. Substance use, including tobacco product and cannabis product use 6. Health care utilization (follow up primary care, urgent care, ED, specialty care, readmission) 7. Financial impact 8. Impact on social network/family
**IMS/CIO/Epi-Aid/Lab-Aid/Chemical Exposure Submission:**	No
**IMS Activation Name:**	Not selected
**Submitted through IMS Clearance Matrix:**	Not selected
**Primary Scientific Priority:**	Not selected
**Secondary Scientific Priority (s):**	Not selected
**Task Force Responsible:**	Not selected
**CIO Emergency Response Name:**	Not selected
**Epi-Aid Name:**	Not selected
**Lab-Aid Name:**	Not selected
**Assessment of Chemical Exposure Name:**	Not selected
**Goals/Purpose**	As a result of the follow-up surveillance, NYC DOHMH will take public health action which may include dissemination of findings to keep the public informed of on-going and evolving risks, both short-term and potentially long-term, of using e-cigarettes and or vape

	products, and support clinicians in decision making (i.e., Health Alert to the network of clinicians throughout NYC, MMWR publication, a Dear Colleague?? letter from the NYC Health Commissioner), Additionally, the findings may inform future policy development regarding the local retail e-cigarette environment.
**Objective:**	Although CDC and state and local health departments are investigating a multi-state outbreak of EVALI, the ongoing health outcomes and impact of EVALI among individuals discharged from the hospital remain unknown. Given reports that some local cases are experiencing persistent symptoms and recurrent healthcare utilization, NYC DOHMH would like to conduct follow-up public health surveillance in collaboration with NYS DOH. This surveillance would include NYC residents who have been discharged from the hospital with EVALI to understand the post-acute health impact and determine if there is a need for longer-term surveillance or modifications to existing acute surveillance infrastructure. The follow-up public health surveillance will focus on describing physical, behavioral, and functional outcomes among NYC residents, as well as ongoing healthcare utilization and other related consequences.
**Does your project measure health disparities among populations/groups experiencing social, economic, geographic, and/or environmental disadvantages?:**	Not Selected
**Does your project investigate underlying contributors to health inequities among populations /groups experiencing social, economic, geographic, and/or environmental disadvantages?:**	Not Selected
**Does your project propose, implement, or evaluate an action to move towards eliminating health inequities?:**	Not Selected
**Activities or Tasks:**	New Collection of Information, Data, or Biospecimens
**Target Populations to be Included/Represented:**	Other - NYC residents discharged from the hospital for EVALI
**Tags/Keywords:**	Tobacco ; Marijuana Smoking ; Acute Lung Injury
**CDC's Role:**	Other - I am a CDC fellow working on this project with NYC DOHMH
**Method Categories:**	Individual Interviews (Qualitative)
**Methods:**	telephone surveys
**Collection of Info, Data or Biospecimen:**	paper survey and then entered into a secure Access Database
**Expected Use of Findings/Results and their impact:**	As a result of the follow-up surveillance, NYC DOHMH will take public health action which may include dissemination of findings to keep the public informed of on-going and evolving risks, both short-term and potentially long-term, of using e-cigarettes and or vape products, and support clinicians in decision making (i.e., Health Alert to the network of clinicians throughout NYC, MMWR publication, a #Dear Colleague# letter from the NYC Health Commissioner), Additionally, the findings may inform future policy development regarding the local retail e-cigarette environment.
**Could Individuals potentially be identified based on Information Collected?**	Yes
**Will PII be captured (including coded data)?**	Yes
**Does CDC have access to the identifiers (including**	No

coded data)?:

Is this project covered by an Assurance of Confidentiality? Yes

Does this activity meet the criteria for a Certificate of Confidentiality (CoC)? No

Is there a formal written agreement prohibiting the release of identifiers? No

Funding

Funding Type	Funding Title	Funding #	Original Budget Yr	# Years Award	Budget Amount
CDC Funding Intramural	EISO funded by CDC		2020	2	

HSC Review

HSC Attributes

Epi-Aids Yes

Regulation and Policy

Do you anticipate this project will need IRB review by the CDC IRB, NIOSH IRB, or through reliance on an external IRB? No

Estimated number of study participants

Population - Children Protocol Page #:

**Population - Minors**	Protocol Page #:
**Population - Prisoners**	Protocol Page #:
**Population - Pregnant Women**	Protocol Page #:
**Population - Emancipated Minors**	Protocol Page #:

Suggested level of risk to subjects

Do you anticipate this project will be exempt research or non-exempt research

Requested consent process waviers

**Informed consent for adults**	No Selection
**Children capable of providing assent**	No Selection
**Parental permission**	No Selection
**Alteration of authorization under HIPAA Privacy Rule**	No Selection

Requested Waivers of Documentation of Informed Consent

**Informed consent for adults**	No Selection
**Children capable of providing assent**	No Selection
**Parental permission**	No Selection

Consent process shown in an understandable language

**Reading level has been estimated**	No Selection
**Comprehension tool is provided**	No Selection
**Short form is provided**	No Selection
**Translation planned or performed**	No Selection
**Certified translation / translator**	No Selection
**Translation and back-translation to/from target language(s)**	No Selection
**Other method**	No Selection

Clinical Trial

**Involves human participants**	No Selection
**Assigned to an intervention**	No Selection
**Evaluate the effect of the intervention**	No Selection
**Evaluation of a health related biomedical or behavioral outcome**	No Selection
**Registerable clinical trial**	No Selection

Other Considerations

**Exception is requested to PHS informing those bested about HIV serostatus**	No Selection
**Human genetic testing is planned now or in the future**	No Selection
**Involves long-term storage of identfiable biological specimens**	No Selection
**Involves a drug, biologic, or device**	No Selection
**Conducted under an Investigational New Drug exemption or Investigational Device Exemption**	No Selection

Institutions & Staff

Institutions

Will you be working with an outside Organization or Institution? Yes

Institution	FWA #	FWA Exp Date	Funding	Funding Restriction Amount
New York City Dept of Hlth & Mental Hygiene	FWA00009459	03/18/27		
New York State Dept of Hlth	FWA00003700	01/26/29		

Institution	Funding Restriction Percentage	Funding Restriction Reason	Funding Restriction has been Lifted
New York City Dept of Hlth & Mental Hygiene			

New York State Dept of Hlth			

Institution	Institution Role(s)	Institution Project Title	Institution Project Tracking #	Prime Institution
New York City Dept of Hlth & Mental Hygiene				
New York State Dept of Hlth				

Institution	Regulatory Coverage	IRB Review Status
New York City Dept of Hlth & Mental Hygiene		
New York State Dept of Hlth		

Institution	Registered IRB	IRB Registration Exp. Date	IRB Approval Status
New York City Dept of Hlth & Mental Hygiene	New York City Dept of Hlth & Mental Hygiene IRB #1	10/12/21	
New York State Dept of Hlth			

Institution	IRB Approval Date	IRB Approval Exp. Date	Relying Institution IRB
New York City Dept of Hlth & Mental Hygiene			
New York State Dept of Hlth			

Staff

Staff Member	SIQT Exp. Date	CITI Biomedical Exp. Date	CITI Social & Behavioral Exp. Date	CITI Good Clinical Practice Exp. Date	Staff Role	Email	Phone	Organization
Achala Talati	n/a	n/a	n/a	n/a	Principal Investigator	atalati@health.nyc.org		New York City Dept of Hlth & Mental Hygiene

Data

DMP

Proposed Data Collection Start Date: 3/2/20

Proposed Data Collection End Date: 2/28/21

Proposed Public Access Level: Restricted

Restricted Details:

Data Use Type:

Data Use Type URL:

Data Use Contact:

Public Access Justification: Information collected by the telephone surveys will be confidential and stored on a secure database and paper copies in a locked cabinet

How Access Will Be Provided for Data: see above

Plans for Archival and Long Term Preservation:

Spatiality

Spatiality (Geographic Locations) yet to be added .....

Dataset

Dataset Title	Dataset Description	Data Publisher /Owner	Public Access Level	Public Access Justification	External Access URL	Download URL	Type of Data Released	Collection Start Date	Collection End Date
Dataset yet to be added...									

Supporting Info

No Supporting Info
