# Supplementary material for: Prospective follow-up of New York City residents with e-cigarette, or vaping product use-associated lung injury—2020–2021
Source: PLoS One. 2025 Apr 30;20(4):e0304918. doi: 10.1371/journal.pone.0304918 (PMC12043135; doi:10.1371/journal.pone.0304918)
Supplement: S3 File — (PDF) [file pone.0304918.s003.pdf]

FOLLOW UP EVALI SURVEILLANCE

CASE INTERVIEW FORM

Round 1

Name of interviewer: _____________________________________

Participant name: _____________________________________

**New York State (NYS) ID number**	
**New York City (NYC) ID number**	

Interview Form

INTERVIEW DETAILS

[TO BE COMPLETED BY HEALTH DEPARTMENT STAFF PRIOR TO INTERVIEW]

**INTERVIEW ATTEMPT INFORMATION**	
**Participant interview status**	○ Completed<br>○ Pending<br>○ Refused<br>○ Proxy refused<br>○ Lost to follow up<br>○ Deceased<br>○ Not a case
**Date/time of attempt 1**	Date:<br><br>Time:
**Date/time of attempt 2**	Date:<br><br>Time:
**Date/time of attempt 3**	Date:<br><br>Time:

Please read the following script if you are able to **reach the participant:**

"Hi, my name is [name] and I'm calling from the New York City Department of Health and Mental Hygiene. I'm calling because you are part of a group of people living in New York City who were hospitalized for a lung injury from vaping. We found out about your illness from the New York State Health Department – you probably spoke to someone from there on the phone when you first got out of the hospital. We would like to learn about your symptoms and experiences since you were discharged from the hospital. This information will help us understand more about the long-term effects of this lung injury on people, which is something that we don't currently know. We think the conversation lasting about 30-60 minutes. Do you have time to speak with me now, or is there another time that would be more convenient?"

[IF YES to speaking now:] *"Great, thank you. We will not share anything you say and all of your responses are confidential. Your responses are very important to us so that we can educate the public and healthcare providers about this type of lung injury. If you need a break at any time during our conversation, just let me know and we can pause and continue at a later time. I will be asking you some questions about sensitive topics. Although I do not directly provide mental health services, I can share resources during the call if you need any help. Do I have your permission to proceed?"*

[IF NO to speaking now:] Ok, when would be a good day and time for me to call you back?

[For people under 18yo:] *"Can I please speak with a parent or guardian to get their permission for us to continue talking?"* Read script to parent/guardian and get consent before proceeding.

□ Yes, participant consent/assent obtained
[□ Yes, parent/guardian consent obtained (for participants <18yo)]
□ No, not okay to proceed

If you reach a **family member**:

"Hi, my name is [name] from the New York City Department of Health and Mental Hygiene. I am trying to reach [participant name]. Is this the best phone number and is [participant name] available?

- This is the best phone number and [participant name] is available: use script above
- This is the best phone number but [participant name] isn't available: "Ok, when would be a good day and time for me to call back?"
- Participant is deceased: "I'm very sorry to hear that. Please accept my condolences. Thank you for your time."

- This isn’t the best phone number but [participant name] is available: “Can you please provide me with the best phone number for reaching [participant name]? Thank you so much, I will try calling [participant name] now.”
- This isn’t the best phone number and [participant name] isn’t available: “Can you please provide me with the best phone number for reaching [participant name]? And do you known the best time for me to try calling [participant name]? Thank you so much, I will try calling [participant name] then.”
