# Supplementary material for: Prospective follow-up of New York City residents with e-cigarette, or vaping product use-associated lung injury—2020–2021
Source: PLoS One. 2025 Apr 30;20(4):e0304918. doi: 10.1371/journal.pone.0304918 (PMC12043135; doi:10.1371/journal.pone.0304918)
Supplement: S1 File — (PDF) [file pone.0304918.s001.pdf]

NEW YORK CITY DEPARTMENT OF HEALTH AND MENTAL HYGIENE
Oxiris Barbot, M.D.
Commissioner

Institutional Review Board
NYC Department of Health and Mental Hygiene
Gotham Center – 31A
42-09 28th Street, 14th Floor
Queens, New York 11101-4132
Tele: 347-396-6118
Fax: 347-396-6087
irbadmin@health.nyc.gov

February 6, 2020

Re: 20-014, Follow-up Surveillance on New York City Residents who have been Discharged from the Hospital for E-cigarette, or Vaping, Product Use Associated Lung Injury (EVALI)
Principal Investigator: Achala Talati, M.D.
This Action: Public Health Surveillance that is Non-Research Determination

Achala Talati
Bureau of Tobacco Control
42-09 28th Street, WS 11-133
Long Island City, NY 11101

Dear Dr. Talati:

The Institutional Review Board (IRB) received your request to conduct the activity, **Follow-up Surveillance on New York City Residents who have been Discharged from the Hospital for E-cigarette, or Vaping, Product Use Associated Lung Injury (EVALI)**. The submission indicated that the Centers for Disease Control and Prevention (CDC) along with state and local health departments were investigating a multi-state outbreak of EVALI. At this time, the Bureau of Tobacco Control proposes to conduct a follow-up to the investigation in collaboration with the New York State Department of Health. The follow up activity will focus on describing the physical, behavioral and functional outcomes related to EVALI among NYC residents.

Based on the information provided, the IRB determined that the activity, as described, meets the definition of public health surveillance as set forth under 45 CFR§102(*l*)(2). The activity is limited to only involve procedures that are necessary to allow a public health authority to identify, monitor, assess, or investigate potential health signals, onsets of disease outbreaks, or conditions of public health importance.

In the event there are plans to use the information generated from this effort to conduct activities that would otherwise be defined as research, please contact the IRB prior to implementation. The IRB will determine if the changes impact the initial categorization.

If you have any questions, please contact the IRB.

Regards,

Diana Wong, Chairperson, Institutional Review Board
New York City Department of Health and Mental Hygiene
